# A Humanized Leucine Zipper-TRAIL Hybrid Induces Apoptosis of Tumors both *In Vitro* and *In Vivo*

**DOI:** 10.1371/journal.pone.0122980

**Published:** 2015-04-07

**Authors:** Dmitri Rozanov, Paul Spellman, Alexei Savinov, Alex Y. Strongin

**Affiliations:** 1 Department of Molecular and Medical Genetics, Oregon Health and Science University, Portland, Oregon, United States of America; 2 Sanford Research, Sioux Falls, South Dakota, United States of America; 3 Sanford-Burnham Medical Research Institute, La Jolla, California, United States of America; Georgia Regents University, UNITED STATES

## Abstract

Evidence suggests that stimulating apoptosis in malignant cells without inflicting collateral damage to the host's normal tissues is a promising cancer therapy. Chemo- and radiation therapies that, especially if combined, induce apoptosis in tumor cells have been used for treating cancer patients for decades. These treatments, however, are limited in their ability to discriminate between malignant and non-malignant cells and, therefore, produce substantial healthy tissue damage and subsequent toxic side-effects. In addition, as a result of these therapies, many tumor types acquire an apoptosis-resistant phenotype and become more aggressive and metastatic. Tumor necrosis factor-Related Apoptosis-Inducing Ligand (TRAIL) has been considered a promising and reliable selective inducer of apoptosis in cancerous cells. TRAIL, however, is not uniformly effective in cancer and multiple cancer cell types are considered resistant to natural TRAIL. To overcome this deficiency of TRAIL, we have earlier constructed a yeast-human hybrid leucine zipper-TRAIL in which the yeast GCN4-pII leucine zipper was fused to human TRAIL (GCN4-TRAIL). This construct exhibited a significantly improved anti-tumor apoptotic activity and safety, but is potentially immunogenic in humans. Here, we report a novel, potent, and fully human ATF7 leucine zipper-TRAIL (ATF7-TRAIL) fusion construct that is expected to have substantially lower immunogenicity. In solution, ATF7-TRAIL exists solely as a trimer with a Tm of 80°C and is active against cancer cells both in vitro and in vivo, in a mouse tumor xenograft model. Our data suggest that our re-engineered TRAIL is a promising candidate for further evaluation as an antitumor agent.

## Introduction

Apoptosis is crucial for normal development and homeostasis in metazoans [1]. Mammals and lower vertebrates have evolved a unique signaling mechanism, termed apoptosis that, under certain circumstances, programs individual cells to die [2]. Alternatively, induction of apoptosis is essential for the elimination of oncogenically transformed cells. Multiple cellular pathways triggering apoptosis are described. Over the years, the two main apoptotic pathways, the extrinsic and intrinsic pathways, have been studied in a great detail. The extrinsic pathway involves the interaction of ligands, including TNF, FasL, and TRAIL, with their respective receptors and the consequential activation of the downstream caspases and Bcl-2 family members [3, 4]. The intrinsic pathway is triggered by internal signals (e. g., DNA damage) that are produced following cellular stress and involves the mitochondria. Initiation of either pathway results in chain-like caspases activation followed by proteolysis of cellular proteins and the degradation of chromosomal DNA [3, 4].

TRAIL is a unique member of the TNF family because it is able to trigger apoptosis in a variety of tumor cells but not in normal cells [5–9]. TRAIL is a type II membrane protein and, similar with TNF-α, TRAIL can be shed from the cell surface membrane to produce a soluble, biologically active form [7]. Expression of TRAIL transcripts has been detected in many human tissues, mostly in the spleen, lung, and prostate [10, 11]. TRAIL forms homotrimers with a stoichiometric zinc atom bound by the cysteine residue of each molecule in the trimeric ligand. Zn stabilizes the TRAIL homotrimer and is essential for its biological activity [12, 13]. TRAIL induces apoptosis by utilizing components of both the extrinsic and intrinsic cellular pathways [14, 15]. In the extrinsic pathway, apoptosis is initiated by the interaction of TRAIL with its respective death receptors, DR4 and DR5. These interactions lead to the receptor trimerization, to the clustering of the receptor’s intracellular death domains (DD), and to the formation of the death-inducing signaling complex (DISC). DISC formation leads to the recruitment of an adaptor molecule, FADD, with the subsequent binding and activation of apical caspase-8 and -10. Activated caspase-8 and -10 then cleave and activate the ‘executioner’ caspases-3 and other downstream caspases, followed by the cleavage of the death substrates and, eventually, cell death.

The TRAIL-induced intrinsic pathway involves the cleavage of the proapoptotic Bcl-2 family member Bid by active caspase-8. Truncated Bid is then translocated to the mitochondria where it promotes the release of cytochrome *c* and SMAC/DIABLO into the cytosol *via* interactions with the proapoptotic proteins Bax and Bak [14–16]. By binding to the adaptor protein APAF-1, cytochrome *c* induces the formation of ‘apoptosome’ that activates caspase-9. In turn, proteolytically active caspase-9 causes activation of ‘executioner’ proteases (caspases-3, -6, and -7) in the presence of dATP, which leads to the cleavage of the death substrates. Anti-apoptotic Bcl-2 family members Bcl-2 and Bcl-X_L_ block cytochrome *c* release and, therefore, are negative regulators of the intrinsic apoptotic pathway [17]. The existence of two signaling apoptotic pathways mediated by TRAIL reveals the existence of two different cell types [18]. In type I cells, the apoptotic pathway is independent of the mitochondria and depends on the caspase-8 activation followed by the activation of effector caspases such as caspase-3. In type II cells, apoptosis is dependent on the amplification of the apoptotic signal *via* the mitochondrial (intrinsic) pathway. Overexpression of the anti-apoptotic Bcl-2 protein does not affect apoptosis in type I cells, but blocks apoptosis in type II cells.

TRAIL is a promising agent for cancer therapy because its effect is independent of the functional status of p53 [19, 20] and because of its ability to induce apoptosis in malignant cells *via* both extrinsic and intrinsic pathways, thus increasing the probability of the apoptotic outcome. There are several strategies to employ TRAIL apoptotic activity for therapeutic applications. TRAIL has been used in clinical trials either as a single agent or in combination with chemotherapeutic drugs [21]. Another strategy to utilize TRAIL antitumor activity is to construct the tumor specific antibody-TRAIL fusion proteins in order to selectively amplify interactions of the TRAIL moiety with the DR4 and DR5 receptors expressed on the surface of cancer cells [22, 23]. In addition, Mitchell et al [24] recently developed a unique approach, in which circulating leukocytes with the adherent E-selectin/TRAIL liposomes were effective in killing cancer cells both *in vitro* with human blood samples and *in vivo*, in the mouse bloodstream.

Because TRAIL is active only as a homotrimer [13], in the previous work we constructed a potent and safe to normal cells TRAIL hybrid, in which the yeast GCN4-pII leucine zipper with the Ile substitutions in the “a” and “d’ positions was fused to human TRAIL (GCN4-TRAIL) [25]. The insertion of the GCN4-pII leucine zipper motif at the N-terminus of TRAIL stabilizes the formation of the TRAIL trimers due to the ability of the Ile residues in the “a” and “d’ positions to drive leucine zipper trimer formation [6, 26]. Because the yeast leucine zipper sequence can be immunogenic in humans, we now replaced the yeast GCN4-pII leucine zipper motif with a human leucine zipper peptide to produce a fully human ATF7-pII leucine zipper-TRAIL (ATF7-TRAIL) chimera that is expected to have low immunogenic potential. ATF7-TRAIL exhibited a potent anti-tumor activity in both cell-based tests and assays in mice, thereby endorsing its further evaluation in preclinical mouse models.

## Materials and Methods

### Antibodies and reagents

All reagents were from Sigma, unless otherwise indicated. Rabbit anti-mouse/rat Asialo GM1 antibodies were from Cedarline. ATPLite reagent was from Perkin-Elmer. Wild type *Pichia pastoris* cells was a gift from Dr. Ilya Tolstorukov. The expression vector pGAPZα and zeocin were from Invitrogen.

### Cells

Human breast carcinoma MDA-MB-231, lung carcinoma SK-MES-1, and prostate carcinoma PPC-1 cells were obtained from ATCC. Human primary hepatocytes were obtained from Lonza and cultured in hepatocyte cell growth medium (Lonza). MDA-MB-231, SK-MES-1, and PPC-1 cells were cultured in Dulbecco's modified Eagle's medium (DMEM) supplemented with 10% FBS (DMEM/FBS) and 10 μg/ml of gentamicin.

### Leucine zipper motifs

The selection of human leucine zipper motifs was based on the prediction of the helical content in the peptide sequences and strength of different interactions between parallel two- or three-stranded leucine zippers by AGADIR1 and bZIP algorithms, respectively [27, 28]. The selected human leucine zipper motifs were derived from matrilins (Mat2 and Mat4). coronin 1A (Cor1A), albumin D-box binding protein (DBP), hepatic leukemia factor (HLF), thyrotroph embryonic factor (TEF), CCAAT/enhancer-binding protein beta, epsilon, and gamma (C/EBP-β, C/EBP-ε, and C/EBP-γ, respectively), cyclic AMP-dependent transcription factor 2 and 7 (ATF2 and ATF7), cyclic AMP-responsive element (CRE)-binding proteins 4, 5, and H (CREB4, CREB5, and CREBH, respectively), X-box binding protein 1 (XBP1), NF-kappa-B essential modulator (NEMO), optineurin (NRP), rho guanine nucleotide exchange factor 7 (β-PIX), TNF receptor-associated factor 1 (TRAF1), and neurabin-I (NRBI) genes.

### Construction of the secretion vector, stability analysis, expression, and purification of TRAIL

The Gln^120^-Gly^281^ portion of the human *TRAIL* gene was linked by the PCR *via* 2–4 amino acid residues linker or without any linker to the modified human leucine zipper motifs where amino acid residues in the “a” and “d” positions of the peptide sequence were substituted by Ile. The leucine zipper-TRAIL fusion constructs were cloned into the pGAPZα plasmid and the resulting expression vectors were transfected into yeast *P*. *pastoris* cells. We analyzed at least 50 clones for each construct for their ability to induce cytotoxicity in the prostate carcinoma PPC-1 cells. For these purposes, TRAIL expressing yeast cells were inoculated into 1 ml of YPD medium supplemented with 100 mM potassium phosphate buffer, pH 7.4 and 100 μg/ml zeocin and grown overnight at 30°C. Next day, equal number of cells for each clone was diluted into 10 ml of YPD supplemented with 100 mM potassium phosphate buffer, pH 7.4 and cells were grown for an additional 48 h. At the end of the growth period, an aliquot of the conditioned media was collected, exchanged for PBS using Micro Bio-Spin columns (BioRad), and used in cytotoxicity assays. The ratio of dead cells as compared to untreated cells was determined by an ATPLite reagent. For TRAIL stability testing, we used fast and simple protocol in which conditioned medium from selected *P*. *pastoris* clones was heated for 20 min at 70°C and then used in cytotoxicity assays. After that, we calculated the residual cytotoxic activity of TRAIL samples after the heat treatment. Expression and purification of leucine zipper-TRAIL fusion proteins was performed as described [25].

### Cell viability assays

Cells grown to subconfluency in wells of a 96-well plate were incubated 24 h with the increasing concentrations of TRAIL in DMEM/FBS. The extent of cell lysis (percent of dead cells) was determined by ATP-Lite reagent (Perkin-Elmer).

### Caspase Assay

The Caspase-Glo 3/7 luminescent assay (Promega) was used to determine caspase-3/7 activity. The resulting luminescence was measured using a plate reader (Tecan).

### Analytical ultracentrifugation and differential scanning calorimetry (DSC)

Sedimentation equilibrium experiments were performed using a ProteomeLab XL-I (BeckmanCoulter) analytical ultracentrifuge. TRAIL samples (0.5, 0.17, and 0.06 mg/ml) in PBS were loaded in the 6-channel equilibrium cells and spun at 20°C for 24 h in an An-50 Ti 8-place rotor at 18,000 rpm. Data were analyzed using HeteroAnalysis software (by J. L. Cole and J. W. Lary, University of Connecticut). DSC of ATF7-TRAIL (0.5 mg/ml in PBS) was performed at a scanning rate of 1 K/min under 3.0 atm of pressure using an N-DSC II differential scanning calorimeter (Calorimetry Sciences).

### Animal Research & Ethics Statement

This study was carried out in strict accordance with the recommendations in the Guide for the Care and Use of Laboratory Animals of the National Institutes of Health. The protocol was approved by the Institutional Animal Care and Use Committee(s) (IACUC) of the Sanford-Burnham Medical Research Institute (Protocol Number: 09–064). Animals were housed in the Sanford Research Laboratory Animal Facility with food and water provided *ad libitum* and monitored daily for their well-being. Humane endpoints (e.g. euthanasia on display moribund characteristics) were in place. All efforts were made to minimize animal suffering during the experiments. At the end of each experiment, animals were euthanized by CO_2_ asphyxiation.

### Tumor xenografts

To test the anti-tumor activity of the ATF7-TRAIL chimera *in vivo*, we used the orthotropic tumor xenograft assays in immunodeficient mice. Female 7–8 week-old NOD.CB17-Prkdcscid/J (NOD-scid) mice were purchased from The Jackson Laboratory, randomized into experimental groups (7–11 mice/group) and subjected to the following experimental treatment protocol. At day 0, to inactivate natural killer (NK) cells all mice received i.v. injection of the asialo-GM1 antibody (0.1mg/animal in 0.2 ml of PBS) followed by the inoculation of MDA-MB-231 cells (1x10^6^ in 0. 1 ml PBS) into the mammary fat pad. The treatment group received injections of purified ATF7-TRAIL (5 mg/kg in 0.2 ml of PBS) i.p. daily for 10 days. The control group received vehicle alone (0.2 ml PBS). To achieve sustained depletion of xenograft-reactive NK cells, second i.v. injection of asialo-GM1 antibody (0.1mg/animal in 0.2 ml of PBS) was administered to animals in all groups at day 4. Mice were sacrificed at day 17 by CO_2_ inhalation. Abdominal cavities and thoraces were cut open, and internal organs were examined for visual signs of organ pathology; then tumors were excised, measured, weighted, and photographed. During the whole length of the treatment, animals were monitored daily for well-being with focus on the treatments-induced toxicity. Specifically, overall appearance, movement activity, ruffing of fur, and stool dryness and uniformity were assessed daily. Food consumption and animal weights were monitored semi-weekly.

## Results

### Design and selection of the ATF7-TRAIL chimera

**Fig 1:**
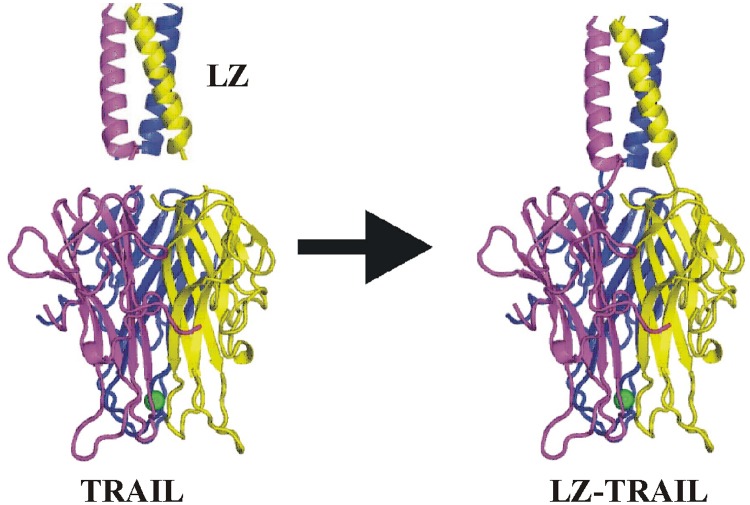
Construction of leucine zipper-TRAIL. LZ, leucine zipper.

**Fig 2:**
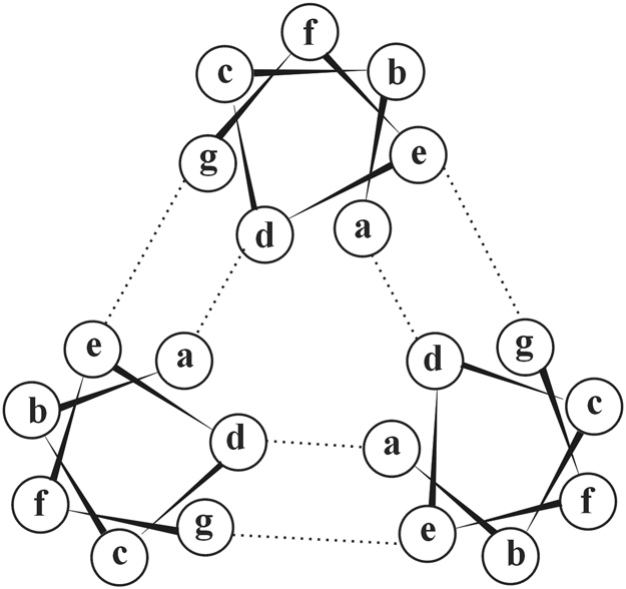
Helical wheel diagram of a trimeric leucine zipper coiled-coil. One heptad repeat is shown down the major axes of the helices. Interhelical hydrophobic a/d and electrostatic e/g interactions in the leucine zipper structure are indicated by dashed lines [26].

We constructed and tested different human leucine zipper motifs with the Ile substitutions at the “a” and “d” positions of the leucine zipper sequence for their ability to stabilize the trimeric structure of TRAIL (Fig 1 and Table 1). It has been published that substitution of all residues in the “a” and “d” positions for Ile resulted in a highly stable GCN4-pII leucine zipper trimeric structure with T_m_>100°C [26]. Because leucine zipper-TRAIL constructs are exogenous soluble proteins, the putative immune response to these fusion proteins can only be mediated by antibodies that recognize protein surface amino acid residues but not by T cells that recognize peptides in the context of MHC class I molecules. We expect that the Ile substitutions in leucine zipper will not induce the significant antibody-mediated immune response because hydrophobic core amino acids in the “a” and “d” positions are directed opposite to the leucine zipper peptide surface (Fig 2). We then determined the effect of the Ile substitutions on the cytotoxic activity of the resulting TRAIL constructs (Table 2). After extensive testing, we selected several leucine zipper-TRAIL-producing *P*. *pastoris* clones for stability analysis (Table 3). Our results showed that the ATF7-pII leucine zipper (VSSIEKKIEEITSQIIQISNEITLIRNEIAQIKQ) with the Ile substitutions at the “a” and “d” positions (underlined in the sequence) was the most efficient in stabilizing the trimeric structure of TRAIL.

**Table 1:** Leucine zipper sequences.

Leucine zipper motifs		heptad I	heptad II	heptad III	heptad IV	heptad V	heptad VI	heptad VII	linker
		abcdefg	abcdefg	abcdefg	abcdefg	abcdefg	abcdefg	abcdefg	
ATF7-pII		VssIekk	IeeItsq	IiqIsne	ItlIrne	IaqIkq			KGSG
ATF2-pII		vqsIekk	IedIssl	IgqIqse	ItlIrne	IaqIkq			
CREB5-pII		VMSIEKK	IEEITQT	IMQIQNE	ISMIKNE	IAQIKQ			
Mat2-pII	EKHDQCK	CENLIMF	QNLANEE	IRKITQR	IEEITQR	IEAIENR	L		GSG
Mat4-pII	CESLVEF	QGRILGA	IESITLN	IAQITAR	IEDIENQ	CESLVEF			GSG
Cor1A-pII	GTPSSDA	ISRIEEE	IRKIQAT	IQEIQQR	IDRIEET	IQAK			
DBP-pII		eke	IalIrqe	IvaIrqe	IshIrav	IsrIqaq	hg		SG
HLF-pII		EKE	ISAIRQE	IADIRKE	IGKIKNI	IAKIEAR	hg		SG
TEF-pII		eke	ItaIrte	IaeIrke	IgkIkti	IskIetk			GSG
C/EBP-β-pII		nleIqhk	IleItae	IerIqkk	IeqIsre	IstIrn			KGSG
TRAF1-pII	RAPCSES	QEELALQ	HFMKEKL	IAEIEGK	IRVIENI	IAVINKE	IEASH		GSG
C/EBP-ε-pII		ileIqqk	IleImae	IerIrsr	IeqItqe	IdtIrn			KGSG
C/EBP-γ-pII	SKqk	IqdIlqr	InqIkee	IerIeak	iklItke	IsvIkd			KGSG
XBP1-pII		mseIeqq	IvdIeee	IqkIlle	IqlIrek	IhgIvve	IqeIrqr		GSG
CREB-H-pII	ETR	ISAITAQ	IQEIQRK	ILHIEKQ	ILSILEQ	IKKIQ			KGSG
CREB4-pII	ESR	IAAISAQ	IQEIQKK	IQEIERH	IISIVAQ	IRQIQ			KGSG
NEMO-pII	gmq	IedIkqq	IqqIeea	IvaIqev	idkIkee	IeqIk			GSG
NRP-pII	DRAV	IKEISEK	IELIeKa	IASIqLQ	IdEIkQT	IAKIEED			RGSG
β-PIX-pII	EEKSLVDT	IYAIKDE	IQEIRQD	NKKIKKS	IEEIQRA	IKDLEKL	IRKI		KGSG
TRAF1-pII	evdcy	rapcses	IeeIalq	IfmIekl	IaeIegk	IrvIeni	IavInke	Ieash	GSG
NRBI-pII	SPSELD	ISKISHK	IKEIQIK	IAVIEAE	IQKIKTK	IQAIENE	K		GSG

Sequences of mutant (-pII) leucine zipper motifs that have been linked *via* linker to the TRAIL sequence are separated by heptad repeats. “a-g” positions in the heptad sequence are shown in the top row. Mutagenized amino acid residues are underlined.

**Table 2:** Ratio of dead cells as compared to untreated cells after TRAIL treatment.

	Leucine zipper motifs								
**V, μl**	CEBP-γ	XBP1	NRP	NEMO	MAT2	Mat4	HLF	DBP	TEF
3	53	14	80	24	47	28	68	83	79
1	24	7	48	18	34	16	28	61	48
	TRAF1	PIX	NRBI	Cor1A	CEBP-β	CEBP-ε	ATF7	CREBH	CREB4
3	3	60	40	15	23	18	85	64	49
1	0	32	15	7	11	6	65	32	17

Conditioned medium from TRAIL-producing *P*. *pastoris* clones was 100-fold diluted in DMEM/FBS. Subconfluent prostate carcinoma PPC-1 in a 96-well plate (100 μl/well) were incubated with 1 and 3 μl of the diluted TRAIL samples for 24 h. At the end of the treatment, the ratio of dead cells was determined by an ATPLite reagent. The conditioned medium obtained from non-transfected yeast cells was not cytotoxic to PPC-1 cells. The cytotoxic activity of the most efficient clones for each TRAIL construct is shown.

**Table 3:** Stability of leucine zipper-TRAIL fusion proteins.

	Leucine zipper motifs								
	NRP	PIX	HLF	DBP	TEF	NRBI	CREB4	ATF7	CREBH
Residual activity, %/10 μl	93	85	97	98	96	38	84	100	78
Residual activity, %/3 μl	72	53	71	79	84	26	51	93	41

The ratio of dead cells was determined as described in the legend for Table 2. Residual cytotoxic activity of the TRAIL samples after 20 min treatment at 70°C was calculated for 3 and 10 μl of 100-fold diluted TRAIL samples.

### ATF7-TRAIL is a stable trimer

**Fig 3:**
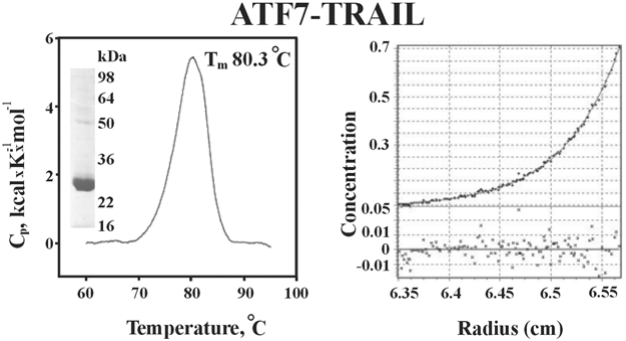
Melting trace (left panel) and sedimentation equilibrium (right panel) of ATF7-TRAIL. Left panel, inset, SDS-PAGE of the FPLC purified ATF7-TRAIL protein. Left panel, the melting temperature (Tm = 80.3°C) value derived from the scan showed that ATF7-TRAIL was correctly folded and very stable. Right panel, data were collected for 0.5 mg/ml of ATF7-TRAIL. Solid line shows the best-fit achieved using a Monomer-Nmer model.

ATF7-TRAIL was purified by Co^2+^-metal chelating chromatography and eluted with a 0 to 25 mM imidazole gradient. The purity of ATF7-TRAIL protein was assessed by SDS-PAGE and ultracentrifugation (Fig 3, *inset*). The fusion of the ATF7 leucine zipper motif to the TRAIL sequence resulted in a highly stable homotrimer. The differential scanning calorimetry indicated that ATF7-TRAIL exists in a single oligomeric form with the melting temperature (Tm) of 80°C (Fig 3, left panel). Our ultracentrifugation analysis also confirmed the purity of our TRAIL sample and indicated that the molecular mass of ATF7-TRAIL was 70.6 kDa (the calculated molecular mass of the ATF7-TRAIL trimer is 69.0 kDa). The sedimentation equilibrium data were analyzed using the Monomer-Nmer model, which showed that ATF7-TRAIL exists solely in its trimeric form (Fig 3, right panel). These data suggested that the ATF7-TRAIL fusion protein was highly purified, properly folded, and stable.

### Potency and safety of the re-engineered ATF7-TRAIL in cell-based tests

**Fig 4:**
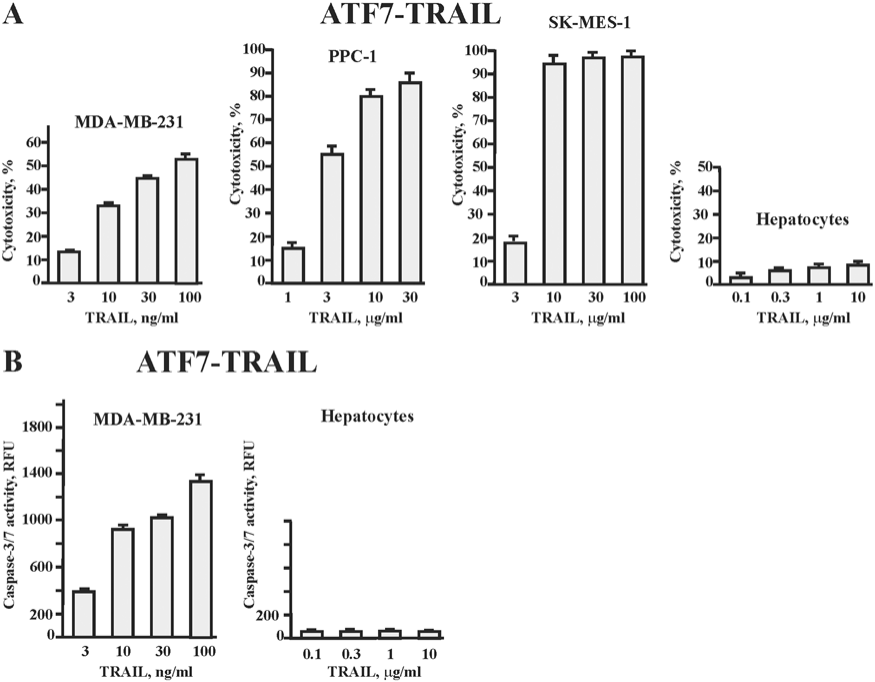
ATF7-TRAIL apoptotic activity. Human breast carcinoma MDA-MB-231, prostate carcinoma PPC-1, lung carcinoma SK-MES-1 cells, and human primary hepatocytes were incubated for 24 h with the increasing concentrations of ATF7-TRAIL. Cytotoxicity (A) and caspase-3/7 activity (B) were determined by ATP-Lite reagent and caspase-3/7 activity assay, respectively. *P* < 0.05.

ATF7-TRAIL at the concentrations as low as 10 ng/ml was highly cytotoxic to breast carcinoma MDA-MB-231, prostate carcinoma PPC-1, and lung carcinoma SK-MES-1 cells (Fig 4A), despite the fact that some of these cells have been considered resistant to TRAIL-mediated apoptosis [29–31]. Beneficially, ATF7-TRAIL did not affect the viability of human primary hepatocytes at a concentration as high as 10 μg/ml. To further confirm that ATF7-TRAIL induced apoptosis in carcinoma cells but not in normal cells, we analyzed the caspases-3/7 activity in TRAIL-treated cells. Consistent with the induction of apoptosis, the low levels of ATF7-TRAIL increased the caspases-3/7 activity in MDA-MB-231 cells. In contrast, ATF7-TRAIL in concentrations as high as 10 μg/ml did not induce any detectable increase in the caspases-3/7 activity in human hepatocytes (Fig 4B).

### Anti-tumor activity of ATF7-TRAIL *in vivo*

**Fig 5:**
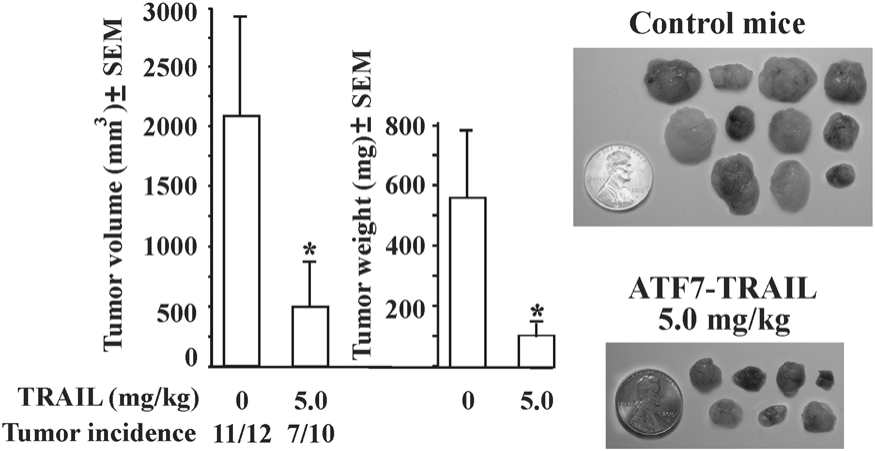
ATF7-TRAIL exhibits a high anti-tumor activity in mice. Left panel, tumor volume and weight of the vehicle-treated and experimental mice that received PBS and 5 mg/kg/day ATF7-TRAIL, respectively. Tumor incidence per group is shown below the bars. *P = 0.004. Right panel, tumors of the control and experimental mice.

We next examined the ability of ATF7-TRAIL to sensitize MDA-MB-231 cells *in vivo* using an orthotopic breast cancer xenograft model in mice (Fig 5). MDA-MB-231 cells were orthotopically xenografted into immunodeficient mice. Animals were randomized into the two groups. The experimental group received an injection of ATF7-TRAIL (5 mg/kg) i.p. daily for 10 days. Control group received PBS alone. We specifically selected a 5 mg/kg dosage of TRAIL because this dosage was used in earlier studies [6, 25]. At a 5 mg/kg concentration, ATF7-TRAIL caused a decrease in tumor incidence and a 4-fold reduction in tumor size relative to control, thus confirming the potency of our TRAIL formulation. Because in the earlier study we have already convincingly demonstrated that GCN4-TRAIL was 50-100-fold more effective in cell-based assays compared with the unaltered TRAIL constructs [25] we selected PBS-treated mice as a control in our animal experiments, rather than unaltered TRAIL-treated mice. In addition, the half-life of GCN4-TRAIL exceeded 1h in mice and, thus, was significantly longer than the half-life of a few minutes reported earlier for the unaltered TRAIL [32]. Based on these data, it was obvious that GCN4-TRAIL should exhibit a significantly better antitumor potency in mice as compared to unaltered TRAIL. Here, we intended to assess the antitumor potency of the ATF7-TRAIL fusion protein and relate it to that of the GCN4-TRAIL chimera having the identical control groups in our both studies. As a measure of response to the treatment, we specifically selected the final tumor volume parameter. This allowed us to compare directly the antitumor potency of ATF7-TRAIL with that of GCN4-TRAIL [25]. In addition, in the vast majority of tumor xenograft studies, tumor growth curves are built based on the typical weekly or bi-weekly measurements. However, these measurements are not applicable for our short-term *in vivo* studies because there would be no statistical significance of our data. As a result, only the final tumor volume was taken into account in our analysis.

Administration of ATF7-TRAIL was considered to be safe as no visible signs of gross organ pathology and/or toxicity were found upon post mortem examinations of experimental animals in all groups. In addition, dynamics of animals weight gain was positive and highly similar in the both control and experimental groups. Moreover, mice did not show any clinical signs of animal distress such as cachexia, cyanosis, dyspnea and ascites, or a lack of mobility and food and water intake at any point during all treatment period.

## Discussion

To facilitate the conversion of the GCN4-TRAIL chimera into a potent anti-tumor agent applicable for cancer therapy, we substituted the yeast GCN4 leucine zipper sequence with several human leucine zipper motifs in which the amino acid residues at the “a” and “d” positions of the coiled-coil heptad repeats of leucine zipper were substituted for Ile (Fig 2). A similar modification in the yeast GCN4 leucine zipper motif resulted in the highly stable leucine zipper trimers (T_m_>100°C) [26]. We hypothesized that the modified human leucine zipper motifs would promote the folding of the hybrid TRAIL construct and stabilize its trimerization. Because the Ile side chains are buried in the protein core, rather than exposed at the leucine zipper surface, we also believed that the Ile substitutions at the “a” and “d” positions would not create an exceedingly strong immunogenic epitopes. Our assumption is supported by the fact that many humanized antibodies that bear mouse sequence in the complementarity determining regions are successfully used in humans without any significant antibody-mediated immune response [33].

As a result of our tests that employed the multiple human leucine zipper sequences, we established that the ATF7-pII leucine zipper peptide promoted the stable trimerization of the resulting leucine zipper-TRAIL hybrids most efficiently. Thus, in solution ATF7-TRAIL existed only as a trimer whose melting temperature exceeded 80°C. These parameters of ATF7-TRAIL were similar to those of the yeast-human GCN4-TRAIL chimera we reported earlier [25]. We also considered a possibility of constructing the ATF2-TRAIL and CREB5-TRAIL fusion proteins in order to compare the stability of all members of cAMP response element (CRE) (5'-GTGACGT[A/G][A/G]-3') binding proteins and to isolate the most stable construct. However, the ATF7, ATF2, and CREB5 leucine zippers are highly homologous. Thus, only 7 and 8 residue positions are non-identical in ATF2 and CREB5 compared to ATF7, respectively (Table 1). From these non-identical positions, only 3 are located in the “e” and “g” positions of either the ATF2 or CREB5 leucine zipper. Amino acid residues in the “e” and “g” positions are involved in electrostatic interactions among leucine zippers in their trimeric structure (Fig 2). Among these “e” and “g” position residues, only one is charged (the third heptad of the CREB5 leucine zipper). Therefore, it is unlikely to expect any significant increase in the stability of the ATF2 and CREB5 leucine zipper trimers relative to that of ATF7. As a result, we have not attempted to construct and test the ATF2-TRAIL and CREB5-TRAIL homologous chimeras.

ATF7 is a ubiquitously expressed transcription factor from the leucine zipper family of DNA binding proteins. ATF7 plays an important functional role in early cell signaling and binds to the CRE sequence present in multiple viral and cellular promoters. ATF7 binds DNA as either a homodimer or heterodimer with other members of the ATF and JUN family of transcription factors. The transcriptional activity of ATF7 is mediated by TAF12, a subunit of the general transcription factor TFIID. ATF7 also interacts with MAPK9. These latter interactions, however, do not result in the ATF7 phosphorylation but they form a docking site for the additional ATF7-associated partners such as JUN [34–36].

To analyze the ATF7-TRAIL apoptotic activity we used caspase 3/7 activation, cell viability, and tumor zenograft assays. Our data demonstrated that the cytotoxic and caspase-3/7-inducing activities and the antitumor potency (as determined by an orthotopic breast cancer xenograft model in immunodeficient mice) of the ATF7-TRAIL and the GCN4-TRAIL constructs were roughly similar [25]. Also, in high similarity to our previous studies with GCN4-TRAIL, animals treated with ATF7-TRAIL in the current study had neither any visible signs of gross organ pathology at sacrifice, nor displayed any clinical or physiological signs of distress during all treatment period. Therefore, we concluded that ATF7-TRAIL treatment was not associated with any major toxic effects *in vivo* and that safety profile of ATF7-TRAIL towards normal tissues is comparable to that of GCN4-TRAIL [25]. Moreover, the low toxicity of ATF7-TRAIL, if any, was additionally supported by its inability to induce caspase-3/7 activity and apoptosis in human primary hepatocytes at the concentration as high as 10 μg/ml.

Because the antitumor activity GCN4-TRAIL and ATF7-TRAIL chimeras was similar, we expected that the half-live of the GCN4-TRAIL and ATF7-TRAIL chimeras are also comparable. Based on these considerations, we did not evaluate ATF7-TRAIL pharmacokinetics in our current study. We believe that our results provide a rationale for the further evaluation of the fully human ATF7-TRAIL antitumor agent using humanized mice with engrafted functional human immune system [37]. We also believe that in order to increase the repertoire of potent and safe TRAIL-based anti-tumor agents it would be beneficial to analyze *in vivo* other fully human leucine-zipper-TRAIL fusion proteins with activity and stability comparable to those of ATF7-TRAIL (Tables 2 and 3). The next-generation anti-tumor TRAIL modifications are clearly needed, especially because of the failure of the recent clinical trials using the recombinant TRAIL and the antibodies targeting the TRAIL death receptors [18]. Combination with chemotherapeutic agents may further enhance the efficacy of ATF7-TRAIL and overcome tumor resistance to TRAIL therapies [18, 21, 38, 39].
